# ADAM10 Regulates Transcription Factor Expression Required for Plasma Cell Function

**DOI:** 10.1371/journal.pone.0042694

**Published:** 2012-08-03

**Authors:** Natalia S. Chaimowitz, Dae-Joong Kang, Lee M. Dean, Daniel H. Conrad

**Affiliations:** Department of Microbiology and Immunology, Virginia Commonwealth University, School of Medicine, Richmond, Virginia, United States of America; University of Miami, United States of America

## Abstract

A disintegrin and metalloprotease 10 (ADAM10) is a key regulator of cellular processes by shedding extracellular domains of transmembrane proteins. We have previously demonstrated that deletion of B cell expressed ADAM10 results in changes in lymphoid tissue architecture and impaired germinal center (GC) formation. In this study, mice were generated in which ADAM10 is deleted in B cells following class switch recombination (ADAM10^Δ/Δ^IgG1-cre^+/−^ mice). Despite normal GC formation, antibody responses were impaired in ADAM10^Δ/Δ^IgG1-cre^+/−^ mice, implicating ADAM10 in post-GC and extrafollicular B cell terminal differentiation. Surprisingly, plasma cell (PC) numbers were normal in ADAM10^Δ/Δ^IgG1-cre^+/−^ mice when compared to controls. However, PCs isolated from ADAM10^Δ/Δ^IgG1-cre^+/−^ mice exhibited decreased expression of transcription factors important for PC function: *Prdm1, Xbp1* and *Irf4.* Bcl6 is a GC transcriptional repressor that inhibits the PC transcriptional program and thus must be downregulated for PC differentiation to occur. Bcl6 expression was increased in PCs isolated from ADAM10^Δ/Δ^IgG1-cre^+/−^ mice at both the mRNA and protein level. These results demonstrate that ADAM10 is required for proper transcription factor expression in PCs and thus, for normal PC function.

## Introduction

Key features of antibody-mediated immune responses are the generation of antigen-specific plasma cells (PCs) and memory B cells. Plasma cells (PCs) are antibody factories and memory B cells can rapidly differentiate into PCs after reencountering antigen. Two general types of PCs are known. Short-lived PCs arise from extrafollicular responses while long-lived PCs are derived primarily from germinal center (GC) B cells [1], [2]. Within GCs, antigen-activated B cells undergo class-switch recombination (CSR), somatic hypermutation (SHM) and affinity maturation [3]. The transition from GC B cell to PC requires changes in the transcriptional program. The transcription factors that are generally required for PC differentiation are B lymphocyte-induced maturation protein 1 (Blimp1), interferon regulatory factor 4 (IRF4) and X-box binding protein 1 (Xbp1) [4]–[7]. GC B cells express Bcl6, a known suppressor of *Prdm1*, the gene encoding Blimp1. Moreover, Blimp1 is also able to repress *Bcl*6. Therefore, as long as Bcl6 is expressed in GC B cells, Blimp1 expression and plasmacytic differentiation are inhibited. If Blimp1 is expressed, however, *Bcl6* will be repressed thus allowing for PC differentiation to occur [8]–[10]. Therefore, downregulation of Bcl6 and Blimp1 upregulation is essential for PC differentiation and optimal humoral responses [1], [2], [11]. Consistent with this idea, study of transgenic mice that constitutively express Bcl6 in B cells showed a decreased number of class-switched PCs [3], [12].

ADAMs (A disintegrin and metalloproteases) are membrane-bound proteins that mediate ectodomain shedding and regulated intramembrane proteolysis (RIP) of transmembrane proteins. Ectodomain shedding releases soluble fragments into the extracellular space, possibly downregulating events that depend on transmembrane receptor expression or activating paracrine signaling by soluble products derived from ADAMs' substrates. ADAMs carry out a wide range of functions, including but not limited to, paracrine signaling, cell adhesion, and intracellular signaling [4]–[7], [13]. ADAM10 is a proteolytically active ADAM family member that is critical for many important biological processes [8]–[10], [14]. Furthermore, as recently described, the intracellular domain of ADAM10 can itself be shed, allowing for the ADAM10 intracellular domain (ICD) to translocate to the nucleus and modulate gene expression [15].

ADAM10 is a key regulator of lymphocyte development [16]. We and others have demonstrated that ADAM10 is essential for T cell and marginal zone B cell development [17], [18]. We recently published that ADAM10 is highly expressed in GC B cells. Interestingly, mice that lack ADAM10 in all peripheral B cells (ADAM10^B−/−^ mice) fail to generate GCs and have severely impaired humoral responses. Furthermore, the defects in antibody production are accompanied by changes in lymphoid architecture [19]. Whether the defects in GC formation and antibody production observed in ADAM10^B−/−^ mice are secondary to the changes in lymphoid architecture or whether ADAM10 plays a role in GC formation and/or antibody production independently of these changes remains to be determined.

In order to investigate the involvement of ADAM10 in PC development and function, ADAM10 was deleted post-isotype switching by crossing ADAM10-floxxed (ADAM10^Δ/Δ^) mice with IgG1-cre^+/−^ mice [20]. In this situation, GCs would form prior to ADAM10 deletion. Here we demonstrate that these recently generated mice showed no alteration in lymphoid architecture and/or GC development. Intriguingly, humoral responses to T-dependent and T-independent antigens were still clearly impaired in ADAM10^Δ/Δ^IgG1cre^+/−^ mice, implicating ADAM10 in B cell terminal differentiation. Furthermore, we show that in spite of normal PC numbers, mRNA expression levels of transcription factors important for PC development, *Prdm1, xbp1* and *Irf4* were altered in PCs isolated from ADAM10^Δ/Δ^IgG1cre^+/−^ mice. In addition, the GC transcription factor Bcl6 was elevated at both the message and protein level. These results demonstrate that ADAM10 is required for proper PC function.

## Results

### Generation of ADAM10^Δ/Δ^IgG1^+/−^ mice

Members of the ADAM family regulate a variety of functions, including, but not limited to, cell migration, proliferation and adhesion [13]. We previously generated mice that lacked ADAM10 in all peripheral B cells (ADAM10^B−/−^ mice) by crossing a transgenic mouse strain containing *lox*P sites surrounding exon 9 of *adam10* (ADAM10^Δ/Δ^) allele with mice expressing Cre recombinase under the control of the CD19 promoter [17]. Study of these mice demonstrated a severe defect in GC formation and changes in lymphoid architecture [19]. In order to determine whether ADAM10 plays a role in PC development or if the impairment in antibody production observed in ADAM10^B−/−^ mice was secondary to changes in architecture, we crossed ADAM10^Δ/Δ^ mice with IgG1-cre transgenic mice and generated ADAM10^Δ/Δ^IgG1-cre^+/−^mice [20]. Previous studies have demonstrated that the IgG1-cre transgene shows specificity for GC B cells, with approximately 75% of GC B cells expressing Cre recombinase. Introduction of the IgG1-cre transgene has been shown to affect IgG1 production; therefore, we also generated controls that have Cre expression but lack ADAM10-floxxed alleles (ADAM10^+/+^IgG1-cre^+/−^ mice). In order to track cells that had undergone cre-mediated recombination, we crossed ADAM10^Δ/Δ^IgG1-cre^+/−^ and ADAM10^+/+^IgG1-cre^+/−^ mice with R26R-EYFP^+^ mice, thus, generating ADAM10^Δ/Δ^IgG1-cre^+/−^R26R-EYFP^+^ (ADAM10^Δ/Δ^IgG1-cre^+/−^YFP^+^) and ADAM10^+/+^IgG1-cre^+/−^YFP^+^ (control) mice that express EYFP transgene following cre-mediated recombination [17].

### Antibody production and GC formation in ADAM10^Δ/Δ^IgG1-cre^+/−^YFP^+^ mice

**Figure 1:**
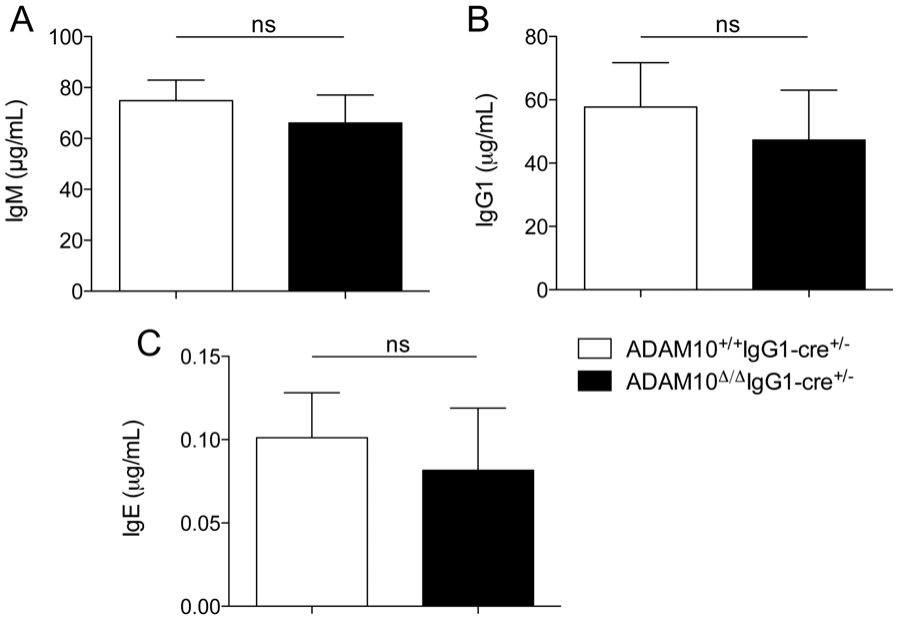
ADAM10^Δ/Δ^IgG1-cre^+/−^ mice have normal basal antibody levels. Serum (A) IgM, (B) IgG1, and (C) IgE were measured by capture ELISA from 8- to 12-wk-old mice. ns: non significant.

Our previous study demonstrated that ADAM10-deletion in all peripheral B cells (ADAM10^B−/−^ mice) led to severe impairments in antibody production, as evidenced from decreased antibody responses to T-dependent antigens. Moreover, GC formation was severely affected in these mice. Furthermore, follicular helper T cell (T_FH_) numbers were also diminished in ADAM10^B−/−^ mice [19]. In order to determine if deletion of ADAM10 in class-switched cells affected basal antibody levels, mice were bled and IgM, IgG1 and IgE levels were measured by ELISA. As depicted in **Figure 1**, no differences were seen between ADAM10^Δ/Δ^IgG1-cre^+/−^YFP^+^ and controls.

**Figure 2:**
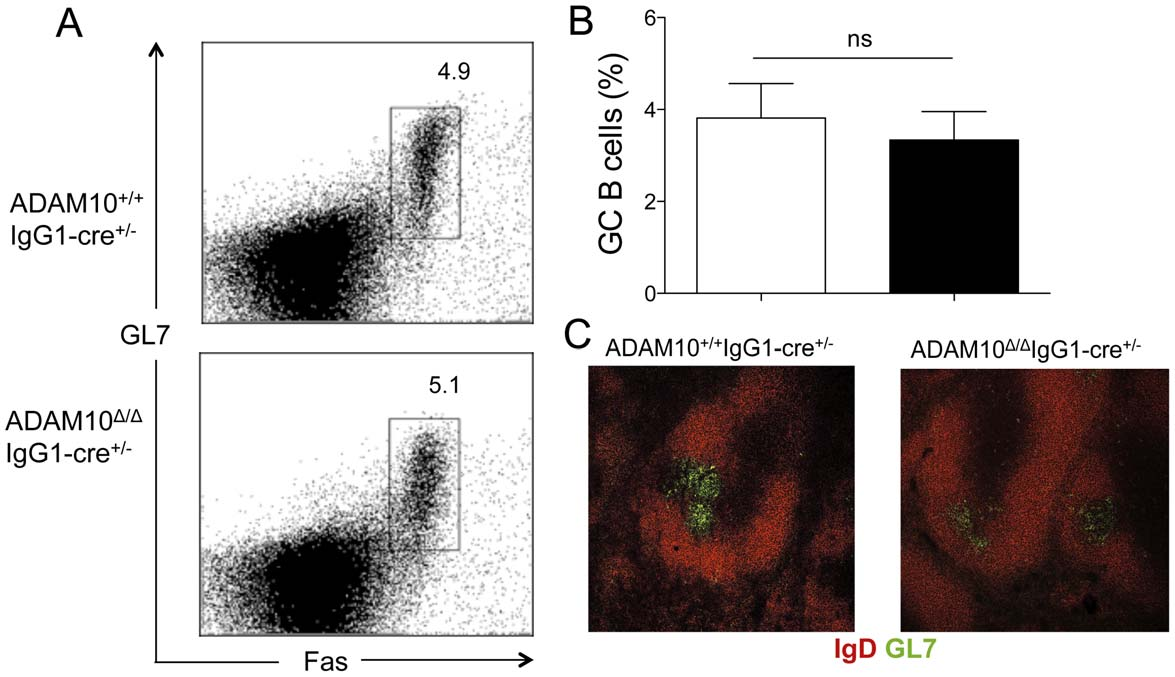
ADAM10^Δ/Δ^IgG1-cre^+/−^ mice have normal germinal center formation. ADAM10^Δ/Δ^IgG1-cre^+/−^ (▪) and controls (□) were immunized with NP-KLH emulsified in alum. Fourteen days post-immunization GC formation was assessed by flow cytometry and immunohistochemistry. (A) Representative dot plot (gated on B220^+^ cells). (B) Frequency of GC B cells of total B cells, representative of 5 mice per group from at least two independent studies. (C) Representative splenic sections stained with GL7 (green) and IgD (red). ns: non significant.

In order to determine whether deletion of ADAM10 within GCs affected GC maintenance and the production of antigen-specific antibodies, ADAM10^Δ/Δ^IgG1-cre^+/−^ mice and controls were immunized with 10 µg of NP-KLH emulsified in alum. The proportion of GC B cells, defined as B220^+^GL7^+^Fas^hi^, was quantified by flow cytometry 14 days post-immunization. The percentage of GC B cells was comparable between ADAM10^Δ/Δ^IgG1-cre^+/−^YFP^+^ and controls (**Figure 2A,B**). Immunohistochemistry analysis also showed normal GCs, defined as clusters of GL7^+^ within B cell follicles (**Figure 2C**). Moreover, the appearance of B cell follicles was comparable to that of controls (**Figure 2C**).

**Figure 3:**
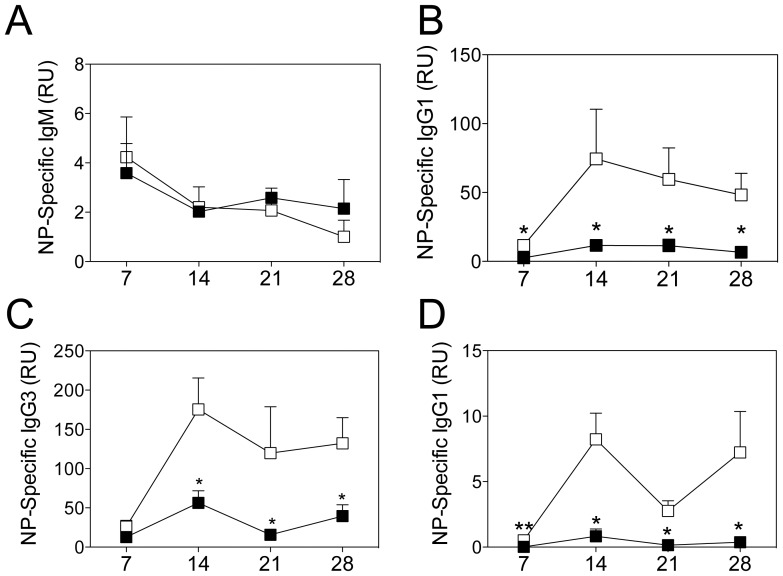
ADAM10^Δ/Δ^IgG1-cre^+/−^ mice show impaired primary antibody responses. ADAM10^Δ/Δ^IgG1-cre^+/−^ (▪) and controls (□) were immunized with a T-dependent antigen, NP-KLH, emulsified in alum (A–B) or a T-independent antigen, NP-LPS (C–D). At the indicated times, serum samples were collected and NP-specific antibodies were measured by capture ELISA. Bars represent the mean ± SE of 5–9 mice per group (*p<0.05, **p<0.01). Data represent results obtained in at least two independent experiments.

Interestingly, despite normal GC B cell numbers, while antigen-specific IgM levels were comparable to control mice (**Figure 3A**), antigen-specific IgG1 was significantly reduced (**Figure 3B**). Moreover, mice were also immunized with 100 µg of NP-LPS, a T-independent antigen. Antibody responses to this antigen were also impaired (**Figure 3C–D**). These results demonstrate a defect in class-switched antibody production in ADAM10^Δ/Δ^IgG1-cre^+/−^ mice.

### Memory B cell development and recall antibody responses in ADAM10^Δ/Δ^IgG1-cre^+/−^YFP^+^ mice

**Figure 4:**
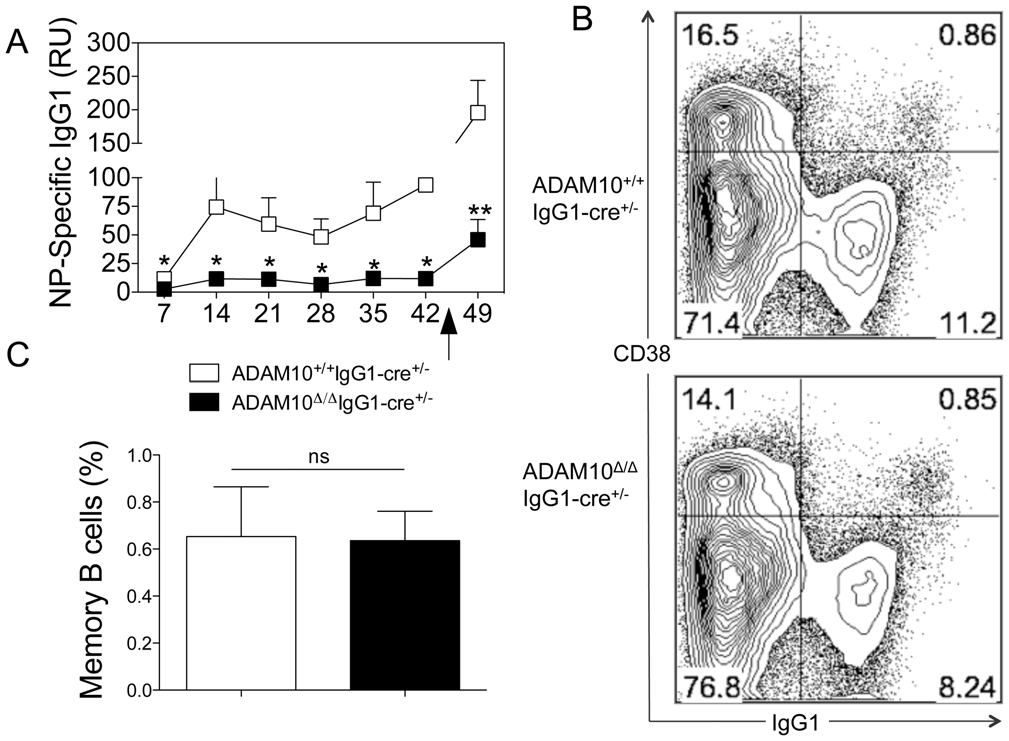
ADAM10^Δ/Δ^IgG1-cre^+/−^ mice show impaired recall antibody responses but normal memory B cell development. (A) ADAM10^Δ/Δ^IgG1-cre^+/−^ (▪) and controls (□) were immunized with NP-KLH emulsified in alum. At the indicated times serum samples were collected and NP-specific antibodies were measured by capture ELISA. Mice were challenged with NP-KLH in alum 6 weeks following primary immunization. Bars represent the mean ± SE of 5–9 mice per group (*p<0.05, **p<0.01). Data represent results obtained in at least two independent experiments. (B–C) ADAM10^Δ/Δ^IgG1-cre^+/−^ (▪) and controls (□) were immunized with NP-KLH emulsified in alum. Fourteen days following immunization, spleens were harvested and memory B cell numbers were analyzed. Memory B cells were defined as B220^+^IgM^lo/−^IgG1^+^CD38^+^ [44].(B) Representative dot plot (gated on B220^+^IgM^lo/−^ cells). (C) Frequency of memory B cells of total B220^+^IgM^lo/−^ cells, representative of 5 mice from at least two independent studies. ns: non significant.

Given that ADAM10^Δ/Δ^IgG1-cre^+/−^ mice showed abnormal primary antibody responses, we sought to determine whether recall antibody responses were also impaired. To this end, mice were immunized with 10 µg of NP-KLH emulsified in alum and boosted 6 weeks later. As demonstrated in **Figure 3**, ADAM10^Δ/Δ^IgG1^+/−^ mice showed decreased production of antigen specific IgG1-antibodies following primary immunization. Moreover, recall responses were also dramatically impaired (**Figure 4A**). Interestingly, when the frequency of memory B cells was analyzed by flow cytometry 14 days following immunization, ADAM10^Δ/Δ^IgG1^+/−^ mice had memory B cells percentages comparable to that of controls (**Figure 4B,C**). These data also suggest that the defects seen in secondary antibody responses result from impaired PC differentiation and/or function.

### Plasma cell development in ADAM10^Δ/Δ^IgG1-cre^+/−^YFP^+^ mice

**Figure 5:**
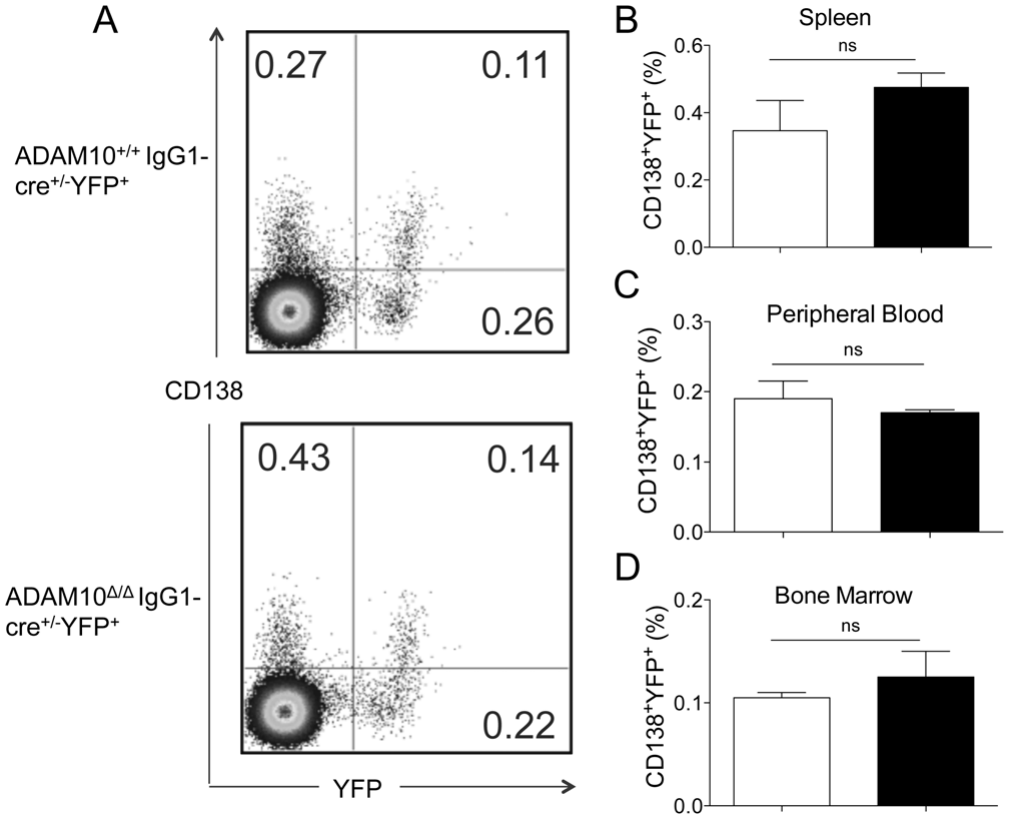
ADAM10^Δ/Δ^IgG1-cre^+/−^ mice have normal plasma cell numbers. ADAM10^Δ/Δ^IgG1-cre^+/−^ (▪) and controls (□) were immunized with NP-KLH emulsified in alum. Twenty-one days following immunization, tissues were harvested and PC numbers were analyzed. Cre-expressing plasma cells were defined as B220^lo/−^CD138^+^YFP^+^. (A) Representative FACS staining of ADAM10^Δ/Δ^IgG1-cre^+/−^ and controls. Frequency of plasma cells of total B220^lo/−^ cells from (B) spleen, (C) peripheral blood and (D) bone marrow. Bars represent the mean ± SE of 4–5 mice per group. Data represent results obtained in at least two independent experiments.

Given that ADAM10^Δ/Δ^IgG1-cre^+/−^YFP^+^ mice had impaired antibody responses but normal GCs, we hypothesized that the defect in antibody production resulted from aberrant PC differentiation. CD138 is marker for antibody-secreting PCs (B220^lo/−^CD138^+^ cells). Accordingly, ADAM10^Δ/Δ^IgG1-cre^+/−^YFP^+^ and control mice were immunized with 10 µg of NP-KLH and the percentage of Cre-expressing PCs (B220^lo/−^CD138^+^YFP^+^ cells) in the spleen, peripheral blood and bone marrow was determined (**Figure 5**) [7]. The gating protocol is depicted in **Figure 5A**. Surprisingly, although ADAM10^Δ/Δ^IgG1-cre^+/−^YFP^+^ mice had markedly impaired antigen-specific IgG1 responses, they had PC percentages comparable to that of controls (**Figure 5B–D**). Previous studies revealed a similar phenotype when humoral responses were studied in B-cell specific XBP1-deficient mice [21]. XBP-1 is a protein involved in ER-stressed and is required for antibody-secretion [21]. These data suggested that ADAM10 might regulate antibody production by modulating XBP-1 expression in PCs.

### Abnormal gene expression in plasma cells isolated from ADAM10^Δ/Δ^IgG1-cre^+/−^YFP^+^ mice

**Figure 6:**
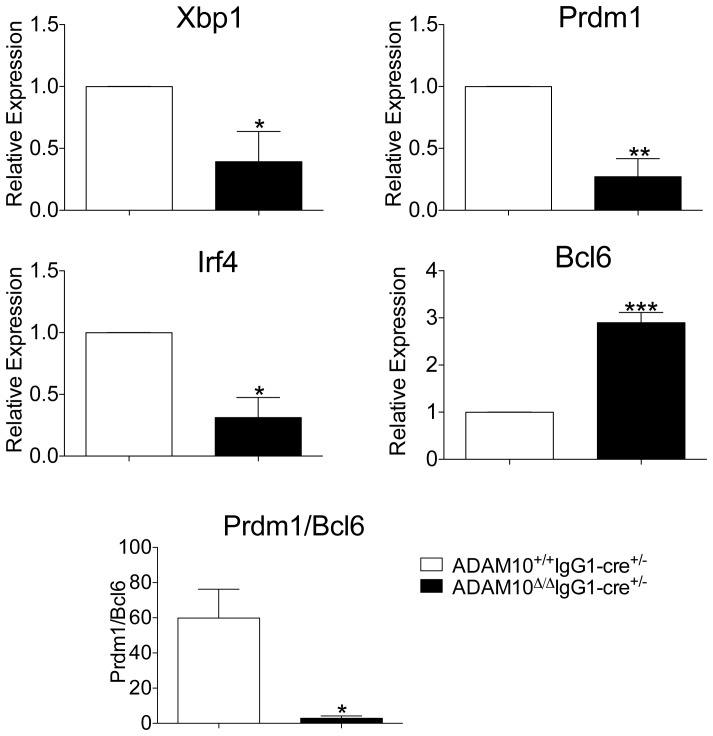
Plasma Cells from ADAM10^Δ/Δ^IgG1-cre^+/−^ mice have altered gene expression. ADAM10^Δ/Δ^IgG1-cre^+/−^ and controls were immunized with NP-KLH emulsified in alum. Twenty-one days following immunization, splenic PCs were isolated via magnetic bead isolation. mRNA was isolated and (A) *Xbp1* (B) *Prdm1*, (C) *Irf4* and (D) *Bcl6* message levels were determined by qPCR. (E) The ratio of *Prdm1* to *Bcl6* was calculated. Bars represent the mean ± SE of 3 independent studies; cells from 3 mice from each genotype pooled in each study. (*p<0.05, **p<0.01, ***p<0.001).

Given that the phenotype observed in ADAM10^Δ/Δ^IgG1-cre^+/−^YFP^+^ mice resembled that of B-cell specific XBP1-deficient mice, *Xbp1* levels were determined in PCs isolated from ADAM10^Δ/Δ^IgG1-cre^+/−^YFP^+^ and control mice. Interestingly, *Xbp1* message levels were significantly reduced when compared to controls (**Figure 6A**). Studies have demonstrated that Xbp1 expression is preceded by the downregulation of Bcl6 and the increased expression of Blimp1 and IRF4 [21]. Thus, the expression of these genes was examined. PCs isolated from ADAM10^Δ/Δ^IgG1-cre^+/−^YFP^+^ mice also showed a reduction in message levels for *Prdm1* (the gene encoding for Blmp1) (**Figure 6B**) and *Irf4* (**Figure 6C**). Moreover, *Bcl6* levels were significantly higher than in control PCs (**Figure 6D**). Even more striking, while PC isolated from controls had ∼60 fold more *Prdm1* message than *Bcl*6, PCs isolated from ADAM10^Δ/Δ^IgG1-cre^+/−^YFP^+^ mice showed only ∼3 fold more *Prdm1* than *Bcl*6 (**Figure 6E**). These results demonstrate that ADAM10 is required for the proper downregulation of *Bcl6* and upregulation of *Prdm1, Irf4* and *Xbp1,* and thus for optimal PC function and production of class-switched antibodies.

**Figure 7:**
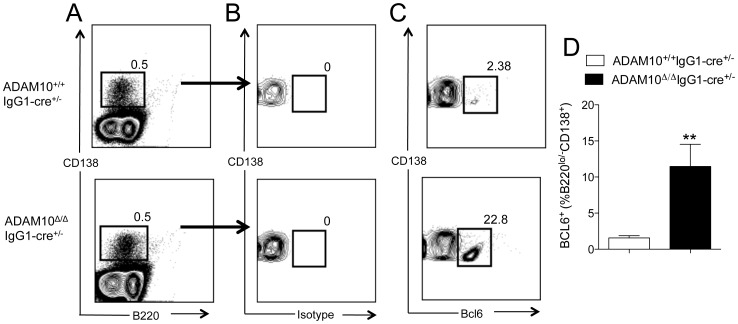
Plasma cells isolated from ADAM10^Δ/Δ^IgG1-cre^+/−^ mice express Bcl6. ADAM10^Δ/Δ^IgG1-cre^+/−^ and controls were immunized with NP-KLH emulsified in alum. Twenty-one days following immunization, splenic plasma cells were analyzed by Bcl6 protein expression via flow cytometry. (A) Plasma cells were defined as CD138^+^B220^lo/−^. After gating on these cells, they were analyzed for (B) non-specific (isotype) or (C) Bcl6-specific staining. (D) Frequency of Bcl6^+^ cells of plasma cells. Bars represent the mean ± SE of 4–5 mice. (**p<0.01).

Consistent with the gene expression results, flow cytometry analysis revealed the presence of a B220^lo/−^CD138^+^Bcl6^+^ population in the spleens of ADAM10^Δ/Δ^IgG1-cre^+/−^ mice following immunization. Splenocytes were isolated and stained for B220 and CD138 (**Figure 7A**). B220^lo/−^CD138^+^ cells were analyzed for non-specific (isotype) or Bcl6-specific staining (**Figure 7B,C**). Quantified results are displayed in **Figure 7D**. These results demonstrate that in the absence of ADAM10, Bcl6 is overexpressed at both the message and protein level.

## Discussion

**Figure 8:**
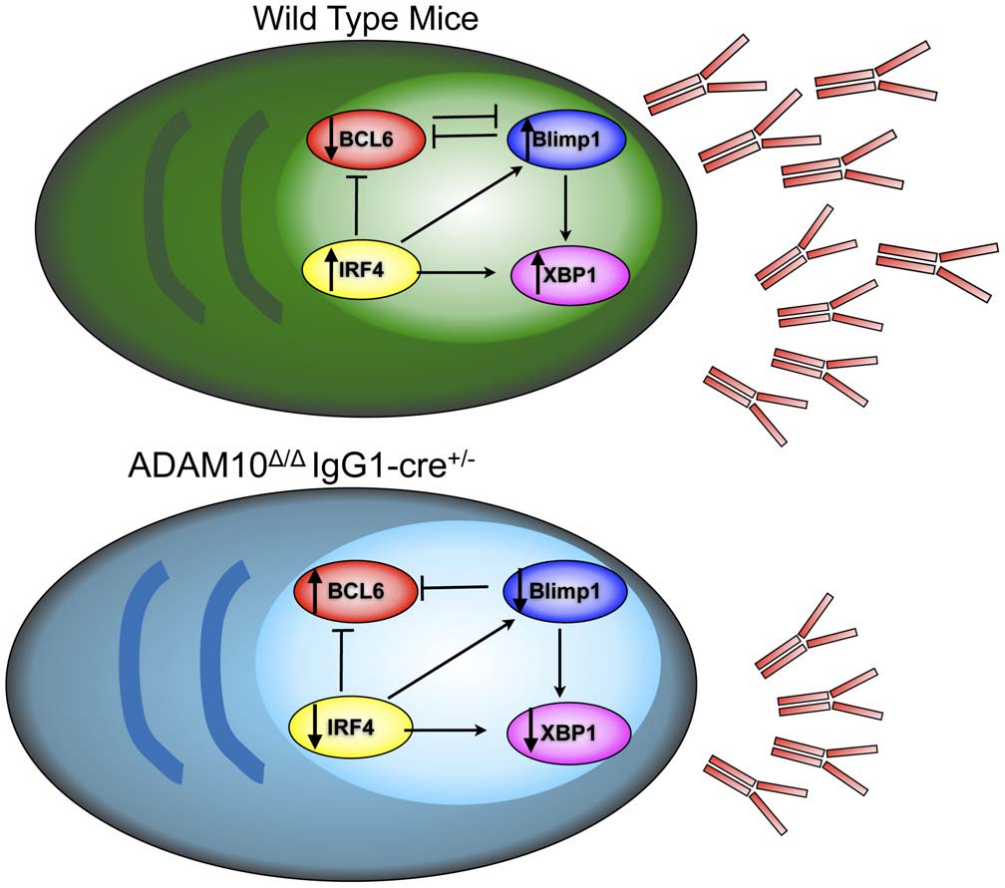
Model. Wild type plasma cells express higher levels of Blimp1, IRF4 and XBP1, while Bcl6 is repressed. This allows for antibody secretion. In the case of ADAM10^Δ/Δ^IgG1-cre^+/−^ mice, Bcl6 levels are higher than seen in wild type. Moreover, Blimp1, IRF4 and XBP1 expression are decreased, leading to impaired antibody secretion.

Members of the ADAM family regulate a wide range of functions, including cell migration, proliferation and adhesion [22]. ADAM10, in particular, has been recently shown to be critical for lymphocyte development through initiation of the canonical Notch signaling pathway [17], [18]. We recently published that ADAM10 is highly expressed in GC B cells. Interestingly, mice that lacked ADAM10 in all peripheral B cells fail to generate GCs and have severely impaired humoral responses. Furthermore, defects in antibody production are accompanied by changes in lymphoid architecture [19]. Here we demonstrate that mice with ADAM10 deletion in class-switched B cells (ADAM10^Δ/Δ^IgG1-cre^+/−^ mice) have normal GC formation and show no changes in splenic architecture. These mice, however, showed reduced production of class-switched antibodies. Given that Cre under the control of the IgG1 promoter is preferentially expressed in class-switched cells, the current study does not allow us to determine the role of ADAM10 in IgM-producing cells. As summarized in **Figure 8**, we have demonstrated that in spite of normal PC percentages, expression levels of proteins important for PC development, *Prdm1, xbp1* and *Irf4* were diminished compared to controls. In addition, the GC transcription factor Bcl6 was not properly downregulated in ADAM10^Δ/Δ^IgG1cre^+/−^ mice. These results demonstrate that ADAM10 is required for the appropriate downregulation of *Bcl6* and upregulation of *Prdm1, Irf4* and *Xbp1*. Subsequently, antibody responses are abnormal in ADAM10^Δ/Δ^IgG1cre^+/−^ mice. Thus, ADAM10 is important for proper PC function.

The transcription factor Bcl6 is necessary for GC formation and B cell proliferation. While it is well established that Bcl6 must be downregulated for class-switched PC differentiation to occur, the factors mediating this event are not fully understood [12]. It has been previously demonstrated that Stat3 activated by IL-21 can trigger Blimp1 expression by competing with Bcl6 for DNA binding sites [9]. Moreover, it has been proposed that B cell receptor (BCR) and CD40 signaling lead to Bcl6 degradation [23]. Recent studies demonstrated that ectopic Stat3 signaling could induce Blimp1 expression, even in the presence of high Bcl6 levels; however, PC differentiation did not proceed until Bcl6 levels were reduced [9]. Here we demonstrate that ADAM10 is important for Bcl6 downregulation and ADAM10 deficiency leads to impaired antibody responses.

Along with defective Bcl6 downregulation observed in ADAM10-deficient PCs, ADAM10 deletion also resulted in decreased levels of *Prdm1*, *Irf4* and *Xbp1.* Blimp1 and IRF4 lead to cell cycle arrest and promote PC differentiation [24], while XBP1 is required for endoplasmic reticulum (ER) expansion and immunoglobulin production and secretion [25]. It has been very well documented that Bcl6 can inhibit Blimp1 [26]. Moreover, Blimp1 and IRF4 permit XPB1 expression [7], [25]. It is possible that ADAM10 is involved in a pathway that controls both Blimp1 and IRF4 expression, and decreased Blimp1 and IRF4 then leads to decreased XBP1 expression.

Bcl6^−/−^ mice respond normally to T-independent antigens, demonstrating that Bcl6 is dispensable for T-independent antibody responses. On the other hand, studies have demonstrated that PC differentiation following T-independent immunizations is dependent on Blimp1 expression [4], [27], [28]. Recent studies have demonstrated that through dual BCR and toll like receptor (TLR) engagement, NP-LPS induces T-independent isotype switching [29]. Interestingly, when ADAM10^Δ/Δ^IgG1cre^+/−^ mice where immunized with NP-LPS, a T-independent antigen that activated B cells through Toll like receptor 4 (TLR4) signaling, impaired antibody production was also evident. It is therefore possible that ADAM10 deletion leads to decreased *Blimp1, Xbp1* and *Irf4* expression in a Bcl6-independent manner. Remarkably, studies of mice deficient in a TLR4 downstream signaling molecule, MyD88, demonstrated that MyD88-deficient B cells also exhibited enhanced Bcl6 expression and diminished Blimp1 expression [30], suggesting that ADAM10 might be involved in TLR4-signaling.

ADAM10 is critical for Notch1 and Notch2 cleavage and the initiation of the canonical Notch signaling pathway [17], [18]. The role of Notch signaling in GC formation and PC differentiation, however, remains controversial. *In vivo* studies of B cell specific RBP-Jκ-deficient mice, a key mediator of Notch signaling, failed to reveal a defect in antibody production [31]. Consistent with this finding, a recently published report demonstrated that B cell-specific Notch2-deficient mice have normal GC formation and normal numbers of splenic PCs [32], [33]. Moreover, mice with constitutively active Notch2 intracellular domain exhibited diminished antibody responses to T-dependent and T-independent antigens and impaired GC formation [34]. On the other hand, *in vitro* studies have demonstrated that Notch signaling enhances B cell activation and supports B cell survival [35]. Moreover, recent studies have shown that Notch signaling synergizes with BCR and CD40 signaling to enhance murine B cell activation [36]. Further studies will be needed, however, in order to elucidate Notch1 and Notch2's role in PC formation and whether the phenotype observed in ADAM10^Δ/Δ^IgG1-cre^+/−^YFP^+^ mice results from impaired Notch signaling.

Another ADAM10 substrate is the IL-6 receptor [37]. Studies have demonstrated that IL-6 signaling can induce Bcl6 expression [38], [39]. In the context of a GC, IL-6 is produced by activated FDCs and promotes SHM, affinity maturation, and CSR [40]. Indeed, IL-6-deficient mice showed decreased SHM following T-dependent immunization [40]. Given that ADAM10 is responsible for the cleavage of the IL-6 receptor (IL-6R) [37], one could speculate that increased IL-6R expression could lead to increased IL-6 signaling, thus, leading to Bcl6 overexpression. Moreover, it is possible that by modulating IL-6R expression levels, ADAM10 can influence SHM and affinity maturation and CSR.

Regulation of Notch signaling is not the only way that ADAM10 is capable of modulating gene expression. Recent experiments have demonstrated that ADAM10 not only mediates RIP, but it is also subject to RIP. ADAM9 and ADAM15 have been identified as the proteases responsible for releasing the ADAM10 ectodomain, while gamma-secretase mediates the release of the ADAM10 intracellular domain (ICD). ADAM10-ICD then translocates to the nucleus and modulates gene expression [15]. Studies have demonstrated that nuclear ADAM10 can interact with androgen receptors and act like a transcription factor [41]. It is thus possible that ADAM10 regulates gene expression and PC differentiation, not by shedding of membrane proteins but through its involvement in gene regulation. ADAM10-ICD regulation of gene expression will require further study.

In conclusion, here we demonstrate that deletion of ADAM10 in class-switched cells does not impair GC formation. However, despite normal PC frequencies, ADAM10^Δ/Δ^IgG1-cre^+/−^YFP^+^ mice showed impaired antibody responses to T-dependent and T-independent antigens. Gene expression analysis demonstrated that PCs isolated from ADAM10^Δ/Δ^IgG1-cre^+/−^YFP^+^ mice expressed lower levels of *Prdm1*, *Irf4* and *Xbp1*. Intriguingly, ADAM10^Δ/Δ^IgG1-cre^+/−^ PCs also expressed 3-fold higher levels of Bcl6. These results demonstrate that ADAM10 is important for the expression of transcription factors that are required for PC differentiation and thus, for optimal antibody production.

## Materials and Methods

### Mice and immunizations

To generate ADAM10^Δ/Δ^IgG1-cre^+/−^YFP^+^ mice, ADAM10^Δ/Δ^YFP^+^ mice were crossed with IgG1-cre^+/−^ mice (Jackson Mice) [19], [42]. ADAM10^+/+^IgG1-cre^+/−^YFP^+^ mice were used as controls. All mouse protocols were approved by the Virginia Commonwealth University Institutional Animal Care and Use Committee. Immunizations comprised of injections of 10 µg 4-Hydroxy-3-nitrophenylacetyl coupled to keyhole limpet hemocyanin at a ratio of 27∶1 (NP_27_KLH, referred to as NP-KLH) in 4 mg of alum or 100 µg of NP-LPS in PBS (Biosearch Technologies). For recall responses to NP-KLH, mice were boosted 6 weeks post-primary immunization with the same dose of antigen as primary immunization.

### Flow cytometry and immunohistochemistry

Single cell suspension of splenocytes were stained as described previously [19]. Abs included anti-mouse unlabeled 2.4G2, FITC conjugated GL7 (GL7); PE-Cy7 conjugated B220 (RA3-6B2) and CD38 (90); allophycocyanin-conjugated GL7 (GL7) and CD138 (281-2); PE-conjugated IgD (11-26c.2a), Fas (Jo2) and PerCP-Cy5.5 conjugated IgM. Flow cytometry analysis was performed using a Canto or Aria II (BD Biosciences), and data analysis was conducted with FlowJo v8.8.7 (Tree Star). Lymphocytes were gated based on FSC vs. SSC. B cell subsets were defined as follows: Plasma cells: B220^lo/−^CD138^+^ [21]; germinal center B cells: B220^+^GL7^+^Fas^hi^ [43]; and memory B cells: B220^+^IgM^lo/−^IgG1^+^CD38^+^ [44]. For immunohistochemistry, spleens were prepared as previously described [19]. Digital images were captured, overlaid, and processed with the Confocal and LCS Lite programs (Leica).

### Cell isolation and Quantitative PCR

Plasma cells were isolated via negative selection (B220^−^DX5^−^ cells) and subsequent positive selection (CD138^+^ cells) using magnetic beads and following manufacturer's instructions (Miltenyi). RNA was extracted and cDNA was generated as previously described [19]. Primers and probes for running a TaqMan quantitative PCR (qPCR) assay were purchased from Applied Biosystems. TaqMan gene expression assays included *Xbp1*: Mm00457357_m1, *Prdm1*: Mm00476128_m1, *Bcl6*: Mm00477633_m1; and *Irf4*: Mm00516431_m1. Reaction parameters were as previously described [17]. Results were analyzed with iQ5 real-time PCR software (version 2.0).

### ELISA

For total IgM and IgG1 ELISAs, samples were serially diluted and added to 96-well plates (50 µL/well) pre-coated with 5 µg/mL of goat-anti IgM and IgG, respectively (Southern Biotech). For standard curve, normal mouse IgM and IgG1 (Southern Biotech) were used. After incubation at 37°C for 1 h, bound Abs were revealed by goat-anti-IgM-AP and goat-anti-IgG1-AP, respectively (Southern Biotech). Total IgE ELISA was carried out as previously described [45]. For antigen-specific ELISAs, ELISA was carried out as described with minor modifications. Plates were coated with NP_14_BSA (Biosearch Technologies)(15 µg/mL in PBS) for samples and with 5 µg/mL of goat-anti Ig (Southern Biotech) in BBS for standard. Standard curves were performed by coating with anti- IgM, anti-IgG1 or IgG3 and adding known amounts of IgM, IgG1 or IgG3, respectively. The values for experimental samples are reflected as relative units (RU) because serum antigen-specific Abs were captured with antigen. The remaining steps were carried out as described.

### Statistical analysis

p-values were calculated using unpaired two-tailed Student's t tests in Graphpad Prism v5. Error bars represent the SEM between samples. p<0.05 is considered statistically significant.
